# Study of Treatment Modalities and Clinical Outcomes of Screen-Detected Cancers at a Tertiary Care Unit in the UK

**DOI:** 10.7759/cureus.84076

**Published:** 2025-05-14

**Authors:** Junaid Zia Hashmi, Muhammad Ali Khattak, Ammarah Ghafoor, Habeeb Abdulrasheed, Jibran Abbasy, Adnan Malik, Amman Malik

**Affiliations:** 1 Breast Surgery, University Hospitals Birmingham NHS Foundation Trust, Birmingham, GBR; 2 Urology, University Hospitals Birmingham NHS Foundation Trust, Birmingham, GBR; 3 Dermatology, Hashmi Hospital Multan, Multan, PAK; 4 Colorectal Surgery, Hull University Teaching Hospitals NHS Trust, Hull, GBR; 5 Surgery, Sandwell and West Birmingham NHS Trust, Birmingham, GBR; 6 Urology, Northampton General Hospital, Northampton, GBR

**Keywords:** ductal carcinoma in situ, ductal carcinoma in situ (dcis), estrogen receptor (er), human epidermal growth factor receptor 2 (her2), invasive breast cancer, magnetic resonance imaging, national health service breast screening program, progesterone receptor (pr), s: magnetic resonance imaging

## Abstract

Introduction

Breast cancer is the most common type of cancer in women worldwide, and early detection plays a key role in improving survival and treatment outcomes. National breast screening programs help identify both invasive and non-invasive cancers, such as ductal carcinoma in situ (DCIS). This study aimed to compare the one-year outcomes of screen-detected invasive breast cancer and DCIS in women diagnosed through a regional screening program.

Methods

A retrospective cohort study was conducted at our tertiary center in the UK. Patients diagnosed with screen-detected breast cancers from January 1, 2023, to December 31, 2024, were followed for one year post-surgery. A total of 216 patients were included: 108 with invasive breast cancer (Group A) and 108 with DCIS (Group B). Data on demographics, tumor characteristics, surgical procedures, postoperative complications, and oncological outcomes were collected using electronic records. Comparative statistical analyses were performed using SPSS version 26 (IBM Corp., Armonk, USA). Chi-square and independent t-tests were used for categorical and continuous variables, and odds ratios (ORs) with 95% confidence intervals were calculated to assess the strength of associations. Statistical significance was set at p < 0.05.

Results

The mean age was similar between groups (Group A: 57.6 ± 10.8 years; Group B: 58.1 ± 11.5 years). Estrogen and progesterone receptor (ER/PR) positivity was high in both groups (70.4% vs. 75%, *p* = 0.431). Human epidermal growth factor receptor 2 (HER2) positivity was more frequent in Group A (17.6% vs 11.1%, *p* = 0.173). Multifocality (24.1% vs 13%, *p* = 0.038), positive margins (17.6% vs 6.5%, *p* = 0.015), and nodal involvement (23.1% vs 0%, *p* < 0.001) were significantly more common in invasive cancers. Postoperative complications (hematoma, wound infection, seroma, flap necrosis) were similar in both groups. However, local recurrence was higher in Group A (9.3% vs 2.8%, *p* = 0.044), and one-year disease-free survival was lower (85.2% vs 97.2%, *p* = 0.002). Chemotherapy was given only to patients in Group A (59.3%).

Conclusion

In our study, we found that screen-detected in situ breast cancer had better short-term outcomes than invasive cancer, with fewer recurrences and higher one-year disease-free survival. Both groups were similar in demographics, but invasive cancer had more multifocality and required more aggressive surgery. Re-excision was more common in the in situ group. The results suggest avoiding overtreatment of DCIS and using risk tools to balance treatment with quality of life. Improving patient education, collaboration, and standardizing surgical decisions is important. The study highlights the need for evidence-based approaches in treatment planning.

## Introduction

According to the World Health Organization, breast cancer is still the most frequent disease among women worldwide and continues to be a major public health concern, accounting for about 685,000 deaths and 2.3 million new cases worldwide in 2020 [1]. The implementation of organized breast screening programs has contributed significantly to early detection and improved clinical outcomes [2]. The National Health Service Breast Screening Programme (NHSBSP) in England invites women aged 50 to 70 years for a 3-yearly mammogram with the aim of early detection of breast cancer. The guidelines and screening modality are different for high-risk patients, in whom screening starts at an early age using magnetic resonance imaging (MRI) as the main modality, with shorter intervals in between [3].

Early breast cancer includes early invasive breast cancer (IBC) and ductal carcinoma in situ (DCIS), where DCIS accounts for 20% of total breast cancer incidences [4]. DCIS has the possible risk of developing into invasive carcinoma if not treated on time; therefore, surgical intervention is mandatory for treating both DCIS and IBC [5]. In addition to surgical management, the IBC often demands more comprehensive medical plans such as chemotherapy, radiotherapy, and hormonal treatment [6]. In the context of recent audits where surgical options such as mastectomy with immediate reconstruction are increasingly being offered, postoperative complications and rates of re-excision due to positive margins continue to vary [7].

Although extensive literature is available from Europe and North America, there is still considerable heterogeneity in treatment modalities and their associated outcomes, particularly in the context of patient comorbidities, tumor biology, and institutional protocols. Comparative data on clinical outcomes between DCIS and IBC, when both are detected via screening, remains limited, especially for surgical complications such as hematoma, wound infection, flap necrosis, and seroma formation [8]. In this study, we seek to address this gap by evaluating the one-year clinical outcomes of screen-detected breast cancers at a tertiary care unit in the UK.

The primary objective of this study is to compare the one-year clinical outcomes, including re-excision, wound complications, and recurrence, between patients diagnosed with screen-detected invasive breast cancer and those with in situ disease. The secondary objective is to examine associations between surgical modality and postoperative outcomes in each group.

## Materials and methods

We conducted a retrospective cohort study to evaluate and compare the 1-year clinical outcome of screen-detected IBC and screen-detected DCIS breast cancers at our center. Data was collected over a period of 2 years, from January 1, 2023, to December 31, 2024, for patients who were diagnosed with screen-detected breast cancers, and they were followed for 1 year at the Department of Breast Surgery, Solihull Hospital, University Hospitals Birmingham. To identify a suitable cohort, an electronic list of all the patients who were diagnosed with breast cancer or DCIS through the screening programme and later on underwent surgery at our center was generated. We searched for codes for mastectomy, mastectomy with immediate reconstruction, wide local excision (WLE), WLE with mammoplasty, and WLE with flap reconstruction. A total of 216 patients were enrolled, with 108 in Group A (screen-detected IBC) and 108 in Group B (screen-detected DCIS).

All patients aged 40 or above with a confirmed diagnosis of screen-detected breast cancer and who underwent surgery at our center with complete records were included. The sampling technique was non-probability, consecutive sampling. Patients with clinically detected breast cancers, missing records, those with a prior history of breast cancer, and patients who declined surgical treatment or were lost to follow-up within one year post-surgery were excluded. Out of the total 310 cases retrieved, 94 were excluded: 34 due to missing information, 33 with clinically identified breast cancer, 17 with a prior history of breast cancer, and 10 lost to follow-up. A thorough examination of medical records and operative notes was conducted to verify that patients met the inclusion criteria.

As this was a retrospective study that did not involve any changes to patient care, it was registered as an audit on Clinical Audits and Registries Management Service (CARMS) and was exempted from Ethical Review Committee (ERC) approval. Following this exemption, data collection was initiated using a specifically designed proforma. A retrospective review of patient medical records, imaging studies, histology reports, surgical operation notes, follow-up letters, and readmission notes was conducted for all the patients who met the inclusion criteria.

Patient demographics (age, menopausal status, family history). Tumor characteristics and histopathological findings (hormone receptor status, human epidermal growth factor receptor 2 (HER2) status, tumor size, multifocality, tumor grade, lymphovascular invasion, and axillary node status) and surgical technique were reviewed from initial assessment forms, clinic letters, histopathology reports, radiology reports, and operation notes. Postoperative outcomes (re-excision for positive margins, hematoma, wound infection, seroma, flap necrosis, and length of hospital stay) and oncologic outcomes (local recurrence, recurrence with distant metastases, disease-free survival at one year, and adjuvant therapies) were reviewed from clinic letters. Surgical interventions for invasive cancers included mastectomy only, mastectomy with immediate free-flap reconstruction, mastectomy with immediate implant reconstruction, WLE, and WLE with mammoplasty.

For in situ cancers, surgical options comprised mastectomy only, mastectomy with immediate free-flap reconstruction, and WLE. Histopathological evaluation categorized tumor grade as I, II, or III and assessed for the presence of lymphovascular invasion and axillary node metastasis. Postoperative outcomes were monitored for complications such as re-excision for positive margins, hematoma, wound infection, seroma, and flap necrosis Oncologic outcomes included assessment of local recurrence, recurrence with distant metastases, disease-free survival at one year, and initiation of adjuvant therapies such as radiotherapy, chemotherapy (for invasive cancers), and hormonal therapy.

Access to the data was restricted to the principal investigator and co-investigators, ensuring confidentiality through the use of coded identifiers. This study did not interfere with the patients' treatment, and the data collected were only used for this analysis. Statistical analysis was performed using SPSS version 26 (IBM Corp., Armonk, USA). Descriptive statistics were used to analyze demographic and clinical characteristics using mean, standard deviation, along with numbers and percentages. Comparative analyses between the two groups employed chi-square tests or Fisher’s exact tests for categorical variables and independent t-tests for continuous variables where appropriate. Odds ratios (ORs) with 95% confidence intervals were calculated to assess the strength of associations. Statistical significance was set at p < 0.05.

## Results

The baseline characteristics of patients with invasive breast cancer (IBC, Group A) and ductal carcinoma in situ (DCIS, Group B) are summarized in Table 1. Both groups included 108 patients. The mean age of patients was comparable between the two groups, with Group A having a mean age of 57.6 ± 10.8 years and Group B 58.1 ± 11.5 years. The majority of patients were post-menopausal, reported in 65 (60.2%) patients in Group A and 63 (58.3%) in Group B. A family history of breast cancer was documented in 24 (22.2%) patients in the IBC group and 19 (17.6%) patients in the DCIS group, as shown in Table 1

**Table 1:** Baseline Characteristics SD: standard deviation; IBC: invasive breast cancer; DCIS: ductal carcinoma in situ; BC: breast cancer

Parameter	IBC Group A (n=108)	DCIS Group B (n=108)
Age (mean ± SD)	57.6 +/- 10.8	58.1 +/- 11.5
Post-menopausal n (%)	65 (60.2%)	63 (58.3%)
Family History of BC n (%)	24 (22.2%)	19 (17.6%)

Regarding the tumor characteristics of patients with IBC (Group A) and DCIS (Group B), estrogen and progesterone receptor (ER/PR) positivity was observed in 76 (70.4%) patients in Group A and 81 (75%) patients in Group B, with no statistically significant difference between the groups. HER2 positivity was found in 19 (17.6%) patients with IBC and in 12 (11.1%) patients with DCIS. Tumor size was recorded only for patients with IBC, with 48 (44.4%) having tumors smaller than 2 cm, 41 (38%) having tumors between 2-5 cm, and 19 (17.6%) with tumors larger than 5 cm. Multifocality was more common in Group A, with 26 (24.1%) patients affected, compared to 14 (13%) in Group B. This difference was statistically significant, indicating that patients with IBC had more than twice the odds of having multifocal disease compared to those with DCIS. Positive surgical margins were significantly more common in Group A, seen in 19 (17.6%) patients compared to seven (6.5%) in Group B. Nodal involvement (≥1 lymph node) was observed in 25 (23.1%) of Group A patients, while no cases were reported in Group B (p < 0.001), highlighting a notable difference between the two groups, as shown in Table 2.

**Table 2:** Tumor Characteristics IBC: invasive breast cancer; DCIS: ductal carcinoma in situ; ER/PR: estrogen receptor/progesterone receptor; HER2: human epidermal growth factor receptor 2; OR: odds ratio; CI: confidence interval; FET: Fisher’s exact test; X2: Chi-square test.

Variable	IBC Group A (n=108)	DCIS Group B (n=108)	p-value (<0.05)	Statistic Test (FET/X2)	OR (95% CI)
ER/PR Positive n (%)	76 (70.4)	81 (75)	0.431	FET	0.85 (0.45–1.62)
HER2 Positive n (%)	19 (17.6)	12 (11.1)	0.173	1.85	1.68 (0.72–3.92)
Tumor Size n (%)					
<2 cm	48 (44.4)	—	—		—
2-5 cm	41 (38)	—	—		—
>5 cm	19 (17.6)	—	—		—
Multifocality n (%)	26 (24.1)	14 (13)	0.038	4.31	2.18 (1.05– 4.56)
Positive Surgical Margins n (%)	19 (17.6)	7 (6.5)	0.015	5.92	2.96 (1.15 7.62)
Nodal Involvement (≥1 node) n (%)	25 (23.1)	0 (0)	<0.001	27.76	—

Regarding surgical interventions and postoperative outcomes for patients with IBC (Group A) and DCIS (Group B). WLE was performed in 59 (54.6%) of Group A patients and 55 (50.9%) of Group B patients, with no significant difference between the groups. Mastectomy was performed in 18 (16.7%) of Group A patients and 16 (14.8%) of Group B patients, showing no significant difference between the two groups. Re-excision was required in 11 (10.2%) of patients in Group A and 19 (17.6%) in Group B, indicating a trend towards a lower rate in Group A, although this was not statistically significant. Hematoma occurred in seven (6.5%) of Group A patients and five (4.6%) of Group B patients, showing no significant difference. Wound infection was noted in nine (8.3%) of Group A and six (5.6%) of Group B patients, with no significant difference between the groups. Seroma occurred in 15 (13.9%) of Group A patients and nine (8.3%) of Group B patients, showing a higher rate in Group A, but the difference was not statistically significant. Flap necrosis was observed in four (3.7%) of Group A patients and two (1.9%) of Group B patients, with no significant difference, as shown in Table 3 below.

**Table 3:** Surgical Interventions and Postoperative Outcomes IBC: invasive breast cancer; DCIS: ductal carcinoma in situ; WLE: wide local excision; OR: odds ratio; CI: confidence interval; X2: Chi-square test.

Parameter n (%)	Group A (n=108)	Group B (n=108)	p-value (<0.05)	Statistic Test X2	OR (95% CI)
WLE	59 (54.6)	55 (50.9)	0.601	0.27	1.20 (0.67–2.15)
Mastectomy only	18 (16.7)	16 (14.8)	0.705	0.15	1.12 (0.52–2.42)
Re-excision needed	11 (10.2)	19 (17.6)	0.104	2.64	0.52 (0.22–1.21)
Hematoma	7 (6.5)	5 (4.6)	0.549	0.34	1.41 (0.42–4.75)
Wound infection	9 (8.3)	6 (5.6)	0.421	0.65	1.50 (0.51–4.47)
Seroma	15 (13.9)	9 (8.3)	0.190	1.71	1.70 (0.68–4.22)
Flap necrosis	4 (3.7)	2 (1.9)	0.405	0.70	1.85 (0.32-10.62)

Considering treatment outcomes in patients with IBC (Group A) and DCIS (Group B). Local recurrence was observed in 10 (9.3%) of Group A patients compared to three (2.8%) of Group B patients, with a statistically significant difference. Distant metastases occurred in seven (6.5%) of patients in Group A, while no cases were reported in Group B. One-year disease-free survival (DFS) was significantly lower in Group A patients, 92 (85.2%), compared to 105 (97.2%) patients in Group B. Adjuvant radiotherapy was administered in 74 (68.5%) of Group A and 65 (60.2%) of Group B patients. Chemotherapy was given to 64 (59.3%) of Group A patients, whereas none of the Group B patients received it. Hormonal therapy was used in 81 (75%) of Group A and 85 (78.7%) of Group B patients, with no significant difference between the groups, as shown in Table 4 below.

**Table 4:** Treatment Outcomes IBC: invasive breast cancer; DCIS: ductal carcinoma in situ; DFS: disease free survival; OR: odds ratio; CI: confidence interval; FET: Fisher’s exact test; X2: Chi-square test.

Parameter n (%)	IBC Group A (n=108)	DCIS Group B (n=108)	p-value (<0.05)	Statistic Test (FET/X2)	OR (95% CI)
Local Recurrence	10 (9.3)	3 (2.8)	0.044	4.04	3.61 (1.03– 13.2)
Distant Metastases	7 (6.5)	0 (0)	—		—
1-year DFS	92 (85.2)	105 (97.2)	0.002	9.65	0.15 (0.04–0.61)
Adjuvant Radiotherapy	74 (68.5)	65 (60.2)	0.202	1.62	1.39 (0.76–2.54)
Chemotherapy	64 (59.3)	0	—		—
Hormonal Therapy	81 (75)	85 (78.7)	0.528	FET	0.84 (0.44–1.60)

## Discussion

In this study, 216 patients were enrolled, equally divided into screen-detected invasive and in situ breast cancer groups. It was observed that both groups shared similar demographic characteristics in terms of age distribution, menopausal status, and family history. Patients in both groups received surgical treatment in the form of either mastectomy only, wide local excision with or without mammoplasty. The DCIS group required additional re-excision procedures more often since their margins were found to be involved. The invasive group experienced most of the local and distant metastasis occurrences. One-year disease-free survival rates stood higher in patients diagnosed within the DCIS group than in those diagnosed with IBC. Hormone receptor status and HER2 positivity were not significantly different between groups. However, a significantly higher proportion of multifocality was seen in the IBC group.

According to the study by Allagoa et al. (2021) from Yale School of Medicine found that screen-detected invasive cancers were more likely to be hormone receptor positive and smaller in size, consistent with our findings on tumor size distribution and ER/PR status. Moreover, the study noted that HER2 positivity in screen-detected cancers did not correlate with increased recurrence at early stages, which aligns with the non-significant HER2 findings in this study [9].

Patients with screen-detected in situ breast cancer obtained better prognostic results, together with reduced recurrence rates alongside less intense surgical operations than patients with invasive breast cancer. Relevant research conducted by da Costa et al. on Canadian patients with ductal carcinoma in situ (DCIS) showed five-year survival reaching 98%, which supports the observed positive effects of screening for in situ lesions in our study [10]. When it comes to the re-excision rate, a Tanzanian multicenter study by Bamusi et al. reported higher rates of re-excision in invasive cancers, in contrast to our finding, where in situ cases had more frequent re-excisions [11]. This discrepancy may be attributed to institutional differences in surgical margins and pathological assessment protocols [12].

Another prospective study conducted in Gambia by Kinteh et al. noted that flap necrosis and postoperative wound complications were more prevalent in in situ surgeries with reconstruction, while in the present study, such complications were marginally more common in invasive cases, although not statistically significant [13]. The observed differences in recurrence and survival could be attributed to the underlying tumor behavior [14]. Invasive cancers exhibit higher proliferation rates and greater potential for lymphovascular invasion, resulting in increased recurrence risk and metastasis [15]. DCIS, by contrast, remains confined within the ductal architecture, leading to better local control and overall prognosis [16]. Multifocality, significantly more prevalent in the IBC group of our study, has been identified as a marker of aggressiveness, as noted by a multicenter Italian study, Keetile et al, which found multifocality to be associated with worse long-term outcomes. The higher disease-free survival and lower recurrence observed in the DCIS group support conservative surgical approaches where appropriate, minimizing overtreatment [17]. 

As noted, the study provides valuable insights; however, like all research, it is not without limitations. Performed in a single tertiary care hospital, even though statistically sufficient, the sample size is too small to identify all complications. Data collection from clinical records may contain elements of observer bias. Evaluation of long-term outcomes was not included. Further studies should focus on integrating genomic and molecular profiling to better understand the biology of screen-detected cancers. Longitudinal multicenter studies with larger sample sizes and diverse populations would improve the external validity of these findings.

## Conclusions

This study demonstrated that screen-detected DCIS was associated with significantly better short-term outcomes compared to screen-detected IBC, with higher one-year disease-free survival and lower rates of local recurrence and distant metastases. Given the higher disease-free survival and lower recurrence in DCIS disease, local protocols should incorporate risk stratification tools to balance oncologic safety with quality of life. The trend of low complication rates and equivalent oncological safety in breast-conserving surgery highlights the importance of early detection of breast cancers, made possible through efficient breast screening programs. In recent times, the adoption of novel screening technologies, alongside advances in artificial intelligence, may further help in achieving the goals of early detection. Efforts should be directed toward improving patient education, enhancing multidisciplinary approaches, and standardizing surgical decision-making pathways. Moving forward, clear pathways for surgery and follow-up care need to be developed to ensure every patient gets the appropriate treatment.

## References

[1] Sharma R, Aashima Aashima, Nanda M (2022). Mapping cancer in Africa: a comprehensive and comparable characterization of 34 cancer types using estimates from GLOBOCAN 2020. Front Public Health.

[2] Coughlin SS, Ekwueme DU (2009). Breast cancer as a global health concern. Cancer Epidemiol.

[3] Assefa AA, Abera G, Geta M (2021). Breast cancer screening practice and associated factors among women aged 20-70 years in urban settings of SNNPR, Ethiopia. Breast Cancer (Dove Med Press).

[4] Ngan TT, Jenkins C, Minh HV, Donnelly M, O'Neill C (2022). Breast cancer screening practices among Vietnamese women and factors associated with clinical breast examination uptake. PLoS One.

[5] Niyaf A, Abdalqader M (2021). Predictors of clinical breast examination and mammography screening uptake among women aged 20-79 years in male’region, maldives. Malay J Pub Health Med.

[6] Bancej C, Decker K, Chiarelli A, Harrison M, Turner D, Brisson J (2003). Contribution of clinical breast examination to mammography screening in the early detection of breast cancer. J Med Screen.

[7] Lin SJ (2008). Factors influencing the uptake of screening services for breast and cervical cancer in Taiwan. J R Soc Promot Health.

[8] Afaya A, Laari TT, Seidu AA, Afaya RA, Daniels-Donkor SS, Yakong VN, Ahinkorah BO (2023). Factors associated with the uptake of clinical breast examination among women of reproductive age in Lesotho: analysis of a national survey. BMC Cancer.

[9] Allagoa DO, Uwaezuoke SC, Kotingo EL (2017). Knowledge, practice and attitude of breast self, clinical breast and mammographic examinations amongst medical doctors in Bayelsa State. Port Harcourt Med J.

[10] da Costa Vieira RA, Biller G, Uemura G, Ruiz CA, Curado MP (2017). Breast cancer screening in developing countries. Clinics (Sao Paulo).

[11] Bamusi MT, Philip NE, Bhat LD (2024). Women's empowerment and its influence on the uptake of breast cancer screening in Tanzania: an analysis of 2022 Tanzania demographic health survey data. BMC Womens Health.

[12] Martín-López R, Jiménez-García R, Lopez-de-Andres A, Hernández-Barrera V, Jiménez-Trujillo I, Gil-de-Miguel A, Carrasco-Garrido P (2013). Inequalities in uptake of breast cancer screening in Spain: analysis of a cross-sectional national survey. Public Health.

[13] Kinteh B, Kinteh SL, Jammeh A, Touray E, Barrow A (2023). Breast cancer screening: knowledge, attitudes, and practices among female university students in The Gambia. Biomed Res Int.

[14] Akter S, Azad T, Rahman MH, Raihan MF (2023). Morbidity patterns and determinants of healthcare-seeking behavior among older women in selected rural areas of Bangladesh. Sulaiman Al Habib Med J.

[15] Agyemang AF, Tei-Muno AN, Dzomeku VM (2020). The prevalence and predictive factors of breast cancer screening among older Ghanaian women. Heliyon.

[16] Rasu RS, Rianon NJ, Shahidullah SM, Faisel AJ, Selwyn BJ (2011). Effect of educational level on knowledge and use of breast cancer screening practices in Bangladeshi women. Health Care Women Int.

[17] Keetile M, Ndlovu K, Letamo G, Disang M, Yaya S, Navaneetham K (2021). Factors associated with and socioeconomic inequalities in breast and cervical cancer screening among women aged 15-64 years in Botswana. PLoS One.

